# Hereditary Ataxias: From Pathogenesis and Clinical Features to Neuroimaging, Fluid, and Digital Biomarkers—A Scoping Review

**DOI:** 10.3390/ijms27020881

**Published:** 2026-01-15

**Authors:** Eugenio Bernardi, Óscar López-Lombardía, Gonzalo Olmedo-Saura, Javier Pagonabarraga, Jaime Kulisevsky, Jesús Pérez-Pérez

**Affiliations:** 1 Movement Disorders Unit, Neurology Department, Sant Pau Hospital, 08025 Barcelona, Spain; ebernardi@santpau.cat (E.B.); olopezl@santpau.cat (Ó.L.-L.); jpagonabarraga@santpau.cat (J.P.); jkulisevsky@santpau.cat (J.K.); 2 Medicine Department, Autonomous University of Barcelona (UAB), 08193 Barcelona, Spain; 3 Center for Biomedical Network Research on Neurodegenerative Diseases (CIBERNED), Instituto de Salud Carlos III, 28029 Madrid, Spain; 4 Sant Pau Biomedical Research Institute (IIB-Sant Pau), 08041 Barcelona, Spain

**Keywords:** biomarker, CANVAS, cerebellar ataxia, FDG-PET, Friedreich’s ataxia, FXTAS, genetic ataxia, hereditary ataxia, MRI biomarker, neurofilament light chain, spinocerebellar ataxia

## Abstract

Hereditary ataxias are a heterogeneous group of disorders with overlapping clinical presentations but diverse genetic and molecular etiologies. Biomarkers are increasingly essential to improve diagnosis, refine prognosis, and accelerate the development of targeted therapies. Following PRISMA-ScR guidelines, we conducted a scoping review of PubMed and complementary sources (2010–2025) to map and describe the current landscape of genetic, imaging, fluid, electrophysiological, and digital biomarkers across the most prevalent hereditary ataxias, including SCA1, SCA2, SCA3, SCA6, SCA7, SCA17, SCA27B, dentatorubral–pallidoluysian atrophy (DRPLA), Friedreich’s ataxia (FRDA), *RFC1*-related ataxia (CANVAS), SPG7, and fragile X-associated tremor/ataxia syndrome (FXTAS). Eligible evidence encompassed observational cohorts, clinical trials, case series, and case reports providing primary biomarker data, with the objective of characterizing evidence breadth and identifying knowledge gaps rather than assessing comparative effectiveness. Across modalities, converging evidence highlights subtype-specific biomarker signatures. MRI volumetry, DTI, and FDG-PET map characteristic neurodegeneration patterns. Fluid biomarkers such as neurofilament light chain are informative across several SCAs and FRDA, while frataxin levels constitute robust endpoints in FRDA trials. Pathology-specific biomarkers such as ataxin-3 are advancing as tools for target engagement and may generalize to future gene-lowering strategies. Electrophysiological and oculographic measures show sensitivity for early disease detection, and wearable technologies are emerging as scalable tools for longitudinal monitoring. This scoping review synthesizes the heterogeneous evidence on hereditary ataxia biomarkers, highlighting multimodal frameworks that link molecular mechanisms with clinical endpoints. Mapping current approaches also reveals substantial variability and gaps across diseases and modalities, underscoring the need for harmonized validation in international multicenter cohorts and systematic integration into future clinical trials to advance precision medicine in hereditary ataxias.

## 1. Introduction

**Figure 1:**
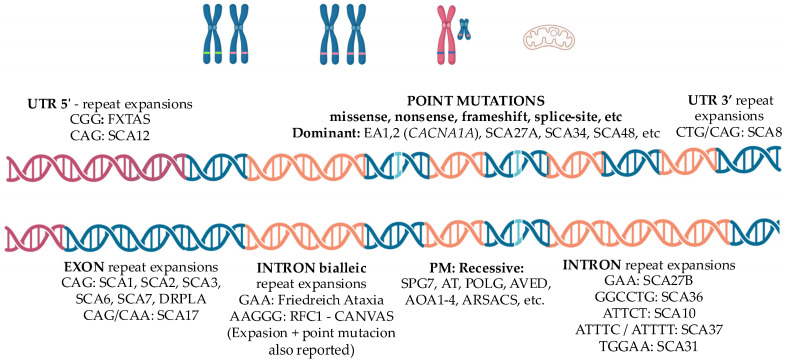
Schematic representation of the main genetic mechanisms underlying hereditary ataxias. All modes of inheritance have been described. Polyglutamine spinocerebellar ataxias (SCAs) are caused by CAG repeat expansions within exons, leading to toxic gain-of-function mechanisms. Other pathogenic variants include tandem repeat expansions located in different genomic regions—5′ untranslated regions (e.g., FXTAS), 3′ untranslated regions (e.g., SCA8), exons (e.g., SCA17), or introns (e.g., SCA27B). Biallelic intronic repeat expansions such as GAA in Friedreich ataxia or AAGGG in CANVAS cause loss-of-function effects. Point mutations, either dominant or recessive, are also implicated. Large deletions, chromosomal rearrangements such as translocations and inversions, as well as mutations in mitochondrial DNA, may also be associated with hereditary ataxia. Red—Untranslated region 5′; Blue—Exon; Orange—Intron. Abbreviations: UTR, untranslated region; PM, point mutation; FXTAS, Fragile X-associated tremor/ataxia syndrome; SCA, spinocerebellar ataxia; EA, episodic ataxia; DRPLA, dentatorubral-pallidoluysian atrophy; CANVAS, cerebellar ataxia, neuropathy and vestibular areflexia syndrome; SPG, spastic paraplegia; AT, ataxia-telangiectasia; POLG, polymerase gamma; AVED, ataxia with vitamin E deficiency; AOA, ataxia with oculomotor apraxia; ARSACS, autosomal recessive spastic ataxia of Charlevoix–Saguenay; CACNA1A, calcium voltage-gated channel subunit alpha1 A.

Hereditary cerebellar ataxias comprise a heterogeneous group of rare, genetically determined disorders with an estimated overall prevalence of 5–6 cases per 100,000 individuals, showing geographical variations depending on the subtype [1]. Their clinical hallmark is the combination of gait and axial imbalance, limb incoordination, dysarthria, and oculomotor disturbances, often accompanied by additional neurological or systemic features [2,3]. They encompass virtually all Mendelian inheritance patterns, as well as mitochondrial transmission, and arise from a wide spectrum of mutational mechanisms—including repeat expansions, exonic variants, intronic and other non-coding region variants, single-nucleotide or small insertion/deletion mutations, among others—underscoring their remarkable genetic heterogeneity (Figure 1). To date, 1098 gene associations have been reported according to the Human Phenotype Ontology database (HP:0001251; https://hpo.jax.org/browse/term/HP:0001251, accessed on 9 September 2025), and this number continues to grow annually. Therefore, a comprehensive review of all hereditary ataxia types exceeds the scope of this article. The pathogenesis of hereditary ataxias is diverse, reflecting multiple molecular mechanisms. In polyglutamine SCAs, the disease arises from a toxic gain of function caused by CAG repeat expansions that encode elongated polyglutamine tracts, leading to misfolded, aggregation-prone proteins, although other types of tandem repeat expansions have also been described. In other forms, intronic or non-coding repeat expansions cause transcriptional dysregulation, as in Friedreich’s ataxia, or RNA-mediated toxicity through sequestration of RNA-binding proteins, as in FXTAS, ultimately resulting in altered gene expression. Additionally, point mutations may reduce protein expression or generate dysfunctional proteins, as in SPG7, thereby affecting mitochondrial homeostasis, ion channel function, DNA repair, and other critical cellular pathways. Altogether, these mechanisms converge on shared pathophysiological pathways leading to cerebellar dysfunction [4,5,6]. From a neuroanatomical standpoint, these disorders primarily affect the cerebellum—either the cerebellar cortex, its deep nuclei, or the spinocerebellar tracts—as well as other cerebellar pathways. Some forms may also involve the dorsal root ganglia, corticospinal tracts, brainstem, and vestibular nuclei, whereas others extend to the basal ganglia and cerebral cortex, accounting for the clinical heterogeneity observed among different ataxia subtypes which is also reflected in the challenges of biomarker development [4,5,6]. Table 1 summarizes key clinical markers that may guide the clinician toward specific subtypes of hereditary ataxias.

In this review, we focus on the most prevalent and clinically relevant subgroups affecting adults that clinicians may find in their clinical practice: spinocerebellar ataxias (SCA1, SCA2, SCA3, SCA6, SCA7, SCA17, SCA27B), dentatorubral–pallidoluysian atrophy (DRPLA), Friedreich’s ataxia (FRDA), cerebellar ataxia with neuropathy and vestibular areflexia syndrome (CANVAS), spastic paraplegia type 7 (SPG7) and fragile X-associated tremor/ataxia syndrome (FXTAS) [1]. Table 2 summarizes genetics and main pathogenesis drivers and clinical features of these conditions.

**The spinocerebellar ataxias (SCAs)** are the most frequent cause of dominantly inherited neurodegenerative ataxia worldwide [1]. Although they share the common substrate of cerebellar degeneration and posterior column involvement, their clinical profiles differ. SCA1, SCA2, and SCA3 (Machado–Joseph disease) are characterized by progressive gait ataxia with pyramidal and/or extrapyramidal features, with cognitive impairment in SCA1 and slow saccades in SCA2 as characteristic features [2,3,7,8,9,10]; SCA3, first described among Portuguese emigrants to Massachusetts [11], up to date, remains the most prevalent SCA globally [1]. SCA6 typically manifests as a relatively “pure” cerebellar syndrome with later onset [12], whereas SCA7 combines ataxia with progressive retinal degeneration leading to blindness [13]. SCA17 may mimic Huntington’s disease, presenting with ataxia, chorea, psychiatric disturbances, and dementia [14]. Dentatorubral–pallidoluysian atrophy (DRPLA) shares overlapping features and clinically manifests with ataxia, seizures, choreoathetosis, myoclonus, and progressive cognitive decline [15,16]. More recently, the late-onset, slowly progressive SCA27B, caused by intronic GAA expansions in FGF14, has been recognized, accounting for up to 20% of patients with late-onset cerebellar ataxia (LOCA) in some series and it is likely to become the most common form of autosomal dominant ataxia worldwide [17]. Despite this heterogeneity, many SCAs share a unifying pathogenic principle: CAG repeat expansions encoding toxic polyglutamine tracts that confer a pathological gain of function to the affected protein [18,19].

**Table 1:** Clinical markers suggestive of specific hereditary ataxias [20,21,22].

Clinical Features	Associated Ataxias
Pure cerebellar ataxia	SCA 5, 6 *, 11, 14, 15, 16, 22
**Ocular/Oculomotor/Vestibular**	
Ophthalmoparesis	SCA 1, 2, 3, 7, SPG7, mitochondrial *, NPC
Slow saccades	SCA 1, 2 *, 3, 7
Oculomotor apraxia	AT, AOA1-4 *, SCA 7
SWJ	FRDA *, *RFC1*, SCA 1, 2, 3
DBN	SCA 1, 3, 6 *, 27B *, *RFC1*
GEN	SCA 1, 3, 6, 27B *, 34, *RFC1*
VOR impairment	SCA 6, 27B, *RFC1* *
Retinitis pigmentosa	SCA 7 *, mitochondrial
Hearing loss	SCA36 *, mitochondrial *
**Neuromuscular**	
Spasticity	SCA 1, 3, 7, FRDA, SPG7 *, ARSACS *, AVED, SYNE1
Peripheral neuropathy	SCA 1, 2, 3, 4, *RFC1* *, FRDA *, AVED, FXTAS, mitochondrial
Myopathy	SCA 28, mitochondrial *
Fasciculations	SCA 36 *
**Movement disorders**	
Chorea	SCA 1, 2, 3, 17 *, 48, DRPLA *, AT
Myoclonus	SCA 2, 17, 19, DRPLA
Tremor	SCA 2 *, 8, 12, 15, 27A
Parkinsonism	SCA 2, 3 *, 12, 17, 48, FXTAS *
Dystonia	SCA 3, 17, AT, DRPLA
**Cognitive/Psychiatric/Other**	
Cognitive impairment	SCA 1 *, 2, 13, 17, 19, 21, 48 *, DRPLA, FXTAS *, mitochondrial
Behavioral disturbances	SCA 1, 17, 48, DRPLA, FXTAS *
Psychosis	SCA 17 *, 27A, DRPLA *
Epilepsy	SCA 10 *, 17, DRPLA, mitochondrial *

* Highly characteristic feature. AOA, Ataxia with oculomotor apraxia (types 1–4); ARSACS, Autosomal recessive spastic ataxia of Charlevoix–Saguenay; AT, Ataxia telangiectasia; AVED, Ataxia with vitamin E deficiency; DBN, Downbeat nystagmus; DRPLA, Dentatorubral–pallidoluysian atrophy; FRDA, Friedreich’s ataxia; FXTAS, Fragile X–associated tremor/ataxia syndrome; GEN, Gaze-evoked nystagmus; NPC, Niemann–Pick disease type C; *RFC1*, Biallelic AAGGG expansion in the *RFC1* gene (CANVAS spectrum); SCA, Spinocerebellar ataxia; SPG7, Spastic paraplegia type 7; SWJ, Square-wave jerks; SYNE1, Autosomal recessive ataxia due to *SYNE1* mutation; VOR, Vestibulo-ocular reflex.

Among **autosomal recessive ataxias (AR)**, Friedreich’s ataxia (FRDA), is the most common form in individuals with onset before 25 years of age. It was originally described by Nikolaus Friedreich in 1863 [23]. It results from GAA intronic expansions in the *FXN* gene, leading to decreased levels of the mitochondrial protein frataxin [24]. Clinically, FRDA course with progressive gait ataxia, dysarthria, and loss of deep tendon reflexes, often accompanied by scoliosis, cardiomyopathy, and diabetes [25,26]. In contrast, among recessive disorders with adult onset, *RFC1*-related ataxia (CANVAS) is now recognized as the most common cause. This syndrome results from biallelic intronic AAGGG repeat expansions in *RFC1* [27,28]. Its core triad—cerebellar ataxia, sensory neuropathy, and bilateral vestibulopathy—is variably associated with chronic cough, reflecting broader autonomic involvement [29,30]. Although SPG7 primarily presents with a spastic paraplegia phenotype, it has also been identified as one of the most frequent causes of hereditary cerebellar ataxia, highlighting the phenotypic overlap between hereditary spastic paraplegias and cerebellar ataxias [31].

The most prevalent **X-linked ataxia** is the Fragile X-associated tremor/ataxia syndrome (FXTAS), typically affecting men over 50 years of age [32,33]. It is caused by premutation-range CGG expansions (55–200 repeats) in the *FMR1* gene (while full expansion > 200 repeats produce Fragile-X syndrome), which lead to toxic RNA gain-of-function, mitochondrial dysfunction, and nuclear inclusions [34,35]. Clinically, FXTAS progresses from tremor and ataxia to neuropsychiatric symptoms and dementia.

Finally, **mitochondrial ataxias** deserve mention as a reminder of the broader spectrum. Conditions such as neuropathy, ataxia, and retinitis pigmentosa (NARP) [36] and Kearns–Sayre syndrome (KSS) [37] arise from mitochondrial DNA deletions, while others, like mitochondrial recessive ataxia syndrome (MIRAS), are due to nuclear mutations in *POLG* that disrupt mitochondrial function [38]. As mitochondrial DNA is maternally inherited, these disorders introduce yet another layer of complexity into hereditary ataxia genetics.

Despite advances in genetic testing, the low prevalence, clinical heterogeneity, and overlapping phenotypes of hereditary ataxias continue to complicate diagnosis and disease monitoring. Age at onset, rate of progression, and associated systemic manifestations vary not only between subtypes but also among individuals carrying the same mutation. These challenges hinder both clinical management and the development of effective therapies. In this context, reliable biomarkers are urgently needed—not only to enable earlier and more accurate diagnosis, but also to provide objective measures of disease severity, risk of phenoconversion, progression, and treatment response, particularly in the emerging era of genetic therapies.

A systematic assessment of available biomarkers—including genetic, imaging, fluid, and electrophysiological measures—represents a critical step toward individualized care and the successful implementation of future clinical trials.

Biomarkers are defined as “a characteristic that is objectively measured and evaluated as an indicator of normal biological processes, pathogenic processes, or pharmacologic responses to a therapeutic intervention” [39]. The U.S. Food and Drug Administration (FDA) expanded this definition through its BEST (Biomarkers, EndpointS, and other Tools) resource, specifying that molecular, histologic, radiographic, or physiologic characteristics may all qualify as biomarkers [40]. Conceptually, an ideal biomarker should be inexpensive, minimally invasive, reproducible, and measurable with high sensitivity and specificity, while also correlating with the underlying pathophysiology in a biologically plausible way [41]. In practice, biomarkers are often combined to increase diagnostic and prognostic yield, particularly in neurology where clinical scales may lack precision and direct brain tissue sampling is rarely feasible [42]. Nonetheless, biomarkers should be considered proxies rather than endpoints in themselves; their ultimate utility lies in predicting, monitoring, or substituting for clinical outcomes of interest.

**Table 2:** Principal genetic biomarkers, pathogenic mechanisms, and distinguishing clinical features in the most prevalent adult-onset hereditary ataxias.

Disorder	Gene	Genetics	Pathogenesis/Topography	Main/Distinguishing Features
**SCA1** [2,43]	*ATXN1* AD	CAG expansion ≥ 39 correlates with onset/severity	Toxic polyglutamine accumulation, Progressive Purkinje cell degeneration (cerebellar cortex). Brainstem, frontotemporal cortex	Hyperreflexia, pyramidal signs, cognitive impairment
**SCA2** [2,44]	*ATXN2* AD	CAG expansion ≥ 33 correlates with onset/severity; intermediate alleles (IA) linked to ALS spectrum	Toxic polyglutamine accumulation. Progressive Purkinje cell degeneration. Brainstem (pontocerebellar nuclei, inferior olive, cranial nerve nuclei) substantia nigra, dorsal root ganglion	Slowness of saccadic eye movements Hyporeflexia Tremor
**SCA3** [2,10]	*ATXN3* AD	Largest CAG expansions among SCAs ≥ 52; predictive of onset	Toxic polyglutamine accumulation. Dentate nucleus, substantia nigra, Brainstem motor nuclei, Basal ganglia.	Progressive external ophthalmoplegia, Parkinsonism, dystonia, pyramidal signs
**SCA6** [2,45]	*CACNA1A* AD	Most stable and smallest CAG-expansion ≥ 20	Toxic polyglutamine accumulation Progressive Purkinje cell degeneration Calcium channel dysfunction	Pure cerebellar ataxia Down beat nystagmus
**SCA7** [46,47]	*ATXN7* AD	Massive unstable CAG expansion ≥ 36	Toxic polyglutamine accumulation Mitochondrial dysfunction Progressive Purkinje cell and retinal photoreceptors degeneration	Vision loss due retinal dystrophy Slowness of saccadic eye movements, oculomotor apraxia Spasticity
**SCA17** [48,49]	*TBP* AD	CAG/CAA expansions ≥ 43 *STUB1* mutations may modulate phenotype in IA (41–42)	Toxic polyglutamine accumulation Purkinje cells, striatum (caudate nucleus and putamen), thalamus, cerebral cortex (mainly frontal, temporal, parietal, and occipital areas), Limbic system (parahippocampus and cingulate gyrus).	HD-Like phenotype Chorea, dystonia, parkinsonism Psychiatric disturbances, cognitive impairment.
**SCA27B** [1,4]	*FGF14* AD	Intronic GAA repeat expansion ≥ 250	Abnormal gene transcription, reduced FGF14 mRNA and protein levels Purkinje and granule cells	Late onset episodic cerebellar ataxia Pure cerebellar ataxia Down beat nystagmus
**DRPLA** [5,48,50]	*ATN1* AD	Massive unstable CAG expansion ≥ 48	Toxic polyglutamine accumulation Dentate nucleus, globus pallidus, caudate, putamen, subthalamic nucleus of Luys, and cerebral cortex	HD-like phenotype: Chorea, parkinsonism, dystonia, cognitive impairment, psychiatric disturbances Juvenile onset: PME
**Friedrich Ataxia** [51]	*FXN* AR	Biallelic GAA intronic repeats expansions ≥ 66 correlate with onset	Decreased gene transcription, Mitochondrial frataxin deficiency Dorsal root ganglia, cerebellar dentate nucleus, Clarke’s column, gracile and cuneate nuclei, and corticospinal tracts.	Areflexia, Babinski sign, square waves, scoliosis, cardiomyopathy, diabetes
***RFC1*** **related ataxia** **CANVAS** [28]	*RFC1* AR	Biallelic pentanucleotide intronic expansions ≥ 400; motif heterogeneity (AAGGG, ACAGG)	RNA expansion–mediated toxicity. Dorsal root ganglia, Purkinje and granular cells of the cerebellum (particularly in the vermis), vestibular ganglia	Sensitive neuronopathy with preserved reflexes Vestibular areflexia Down beat nystagmus Spasmodic dry cough
**SPG7** [52]	*SPG7* AR	Biallelic pathogenic variants (point mutations)	Mitochondrial dysfunction due to paraplegin deficiency Corticospinal tracts, cerebellar (dentate nucleus), brainstem, optic nerve neurons	Cerebellar ataxia (predominant in 1/3 cases) Spastic paraplegia Ophthalmoplegia, optic neuropathy, dystonia
**FXTAS** [35,53]	*FMR1* X-linked	Premutation (55–200 CGG) (>200 CGG lead to X fragile syndrome) in 5′UTR	Predominant astroglial RNA expansion–mediated toxicity White matter of middle cerebellar peduncle, cerebellum, hippocampus, striatum, cortex, corpus callosum	Postural and intentional tremor, parkinsonism, cognitive impairment, neuropsychiatric disturbances Females: milder phenotype, premature ovarian failure.

CANVAS, Cerebellar Ataxia with Neuropathy and Vestibular Areflexia Syndrome; DRPLA, Dentatorubral–Pallidoluysian Atrophy; FRDA, Friedreich’s Ataxia; FXTAS, Fragile X–Associated Tremor/Ataxia Syndrome; HD-Like, Huntington’s disease–like; PME: Progressive myoclonic epilepsy, SCA1, Spinocerebellar Ataxia Type 1; SCA2, Spinocerebellar Ataxia Type 2; SCA3, Spinocerebellar Ataxia Type 3 (Machado–Joseph Disease); SCA6, Spinocerebellar Ataxia Type 6; SCA7, Spinocerebellar Ataxia Type 7; SCA17, Spinocerebellar Ataxia Type 17; SCA27B, Spinocerebellar Ataxia Type 27B; SPG7, Spastic Paraplegia Type 7; UTR, Untranslated Region.

## 2. Methods

**Scheme 1:**
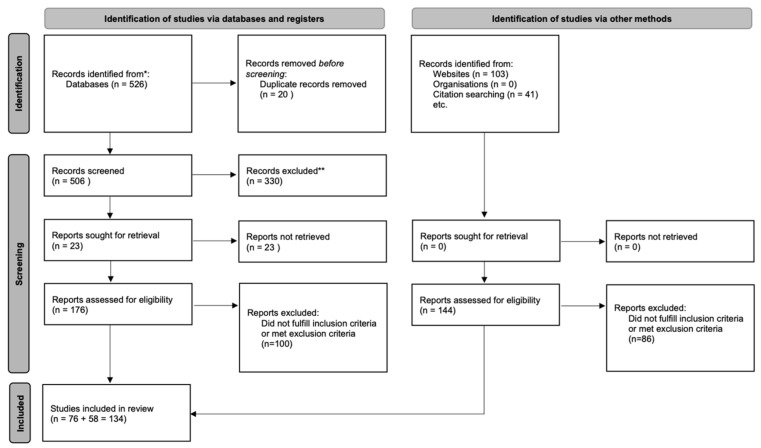
PRISMA 2020 flow diagram summarizing study identification, screening, eligibility, and inclusion * PubMed, Web of Science, Scopus, Cochrane Library ** Did not meet inclusion/exclusion criteria.

A scoping literature search was conducted following the PRISMA-ScR guidelines [54] to identify original studies investigating biomarkers in the more prevalent adult-onset hereditary ataxias. The search aimed to map the breadth and characteristics of available evidence rather than evaluate comparative effectiveness. PubMed, Web of Science, Scopus, and the Cochrane Library were systematically queried using a combination of Medical Subject Headings (MeSH) and free-text terms targeting the following disorders: spinocerebellar ataxias (SCA) 1, 2, 3, 6, 7, 17, 27B; dentatorubral–pallidoluysian atrophy (DRPLA); Friedreich’s ataxia (FRDA); *RFC1*-related ataxia, cerebellar ataxia, neuropathy and vestibular areflexia syndrome (CANVAS); spastic paraplegia type 7 (SPG7); and fragile X–associated tremor/ataxia syndrome (FXTAS). The query included terms referring to imaging, fluid, electrophysiological, and digital biomarkers. The complete search strategies for each database are reported in Scheme 1. Across all databases, the search yielded 526 records as of 9 September 2025.

All retrieved records were uploaded to Rayyan QCRI, an online platform designed to support transparent article screening. Fourteen duplicate entries were removed automatically or manually, resulting in 506 unique articles for further evaluation.

Two reviewers screened titles and abstracts for relevance according to eligibility criteria defined through the Population–Concept–Context (PCC) framework. In this review, the PCC approach reflected our focus on adult hereditary cerebellar ataxias, biomarker-related concepts across multiple modalities, and evidence generated in clinical or translational human studies without restrictions on design. Screening discrepancies were resolved by a third reviewer with experience in neurodegenerative disorders and biomarker research. The screening process was conducted while blinded to each other’s decisions.

Of the 506 unique records, 76 articles met eligibility criteria after full-text screening. An independent citation search then identified an additional 58 relevant studies, yielding a final total of 134 works included in the review.

### 2.1. Inclusion Criteria

Only Case Reports, Clinical Studies, Clinical Trials, Multicenter Studies, Observational Studies, Randomized Controlled Trials published after 2010 were considered. Articles hat to focus on SCA1, SCA2, SCA3, SCA6, SCA7, SCA17, SCA27B, DRPLA, FRDA, *RFC1*-related ataxia (CANVAS), SPG7 or FXTAS, be published material in peer-reviewed journals, in English.

### 2.2. Exclusion Criteria

Articles published before 2010; studies using cellular or animal models; conference abstracts or other non–peer-reviewed sources; publications in languages other than English; studies focused on non-neurological biomarkers; and studies exclusively involving pediatric populations were excluded. However, mixed-age cohorts that included adolescents or young adults were retained (e.g., Friedreich ataxia).

After applying these criteria, 330 articles were excluded. A total of 176 studies were retained for full-text review and data extraction, 76 articles passing this step. Finally, manual bibliographical search revealed 48 more eligible papers, for a total of 124 papers.

## 3. Results and Discussion

### 3.1. Imaging Biomarkers

**Figure 2:**
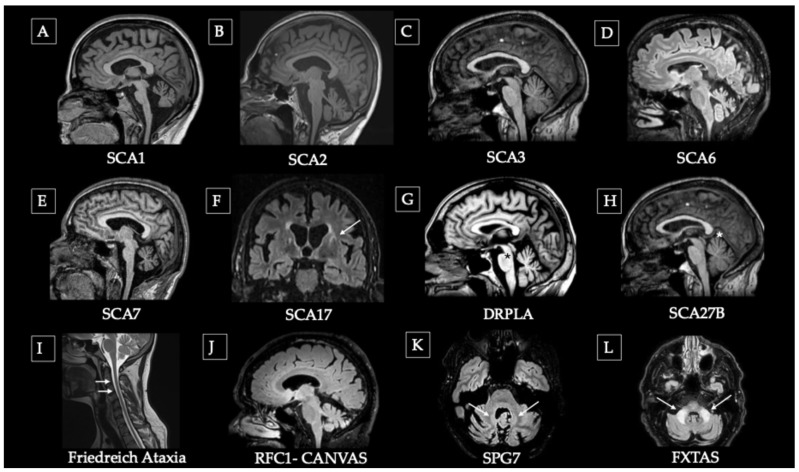
Representative structural MRI patterns in main hereditary ataxias. (**A**–**C**) Olivo-ponto-cerebellar atrophy (OPCA) pattern: (**A**) advanced SCA1, (**B**) moderate SCA2, and (**C**) early SCA3. (**D**) Pure cerebellar atrophy in SCA6. (**E**) Pontocerebellar atrophy pattern. (**F**) SCA17 showing striatal atrophy (caudate) and putaminal rim hyperintensity (arrow). (**G**) DRPLA with pontine hypointensity (asterisk) and cerebellar atrophy. (**H**) SCA27B with mild superior vermian atrophy (asterisk). (**I**) Friedreich’s ataxia showing cervical spinal cord atrophy (arrows) due to ganglionic degeneration with preserved cerebellum. (**J**) CANVAS (biallelic *RFC1* expansion) with mild-to-moderate cerebellar atrophy. (**K**) SPG7 Ataxic variant showing dentate nucleus hyperintensity (arrows), and cerebellar atrophy. (**L**) FXTAS with hyperintensity of the middle cerebellar peduncles (arrows).

Within neuroimaging biomarkers, most studies have focused on structural MRI, ranging from early descriptive clinical reports to longitudinal cohort studies assessing disease progression. Hereditary ataxias may present with normal MRI findings, particularly those related to channelopathies such as episodic ataxia. However, most forms are neurodegenerative and are characterized by distinctive and evolving patterns of atrophy: olivopontocerebellar atrophy in SCA1, SCA2, SCA3, and SCA7; predominant cerebellar atrophy in SCA6; and spinal cord atrophy in Friedreich’s ataxia [55,56]. Additional imaging signatures may also occur, as certain patterns—such as the Hot Cross Bun sign (HCBS), traditionally associated with multiple system atrophy—have been reported in other genetic ataxias, particularly SCA2 and SCA34 [57,58,59], as well as white matter hyperintensities observed in FXTAS with the classic hyperintensity of the medium cerebellar peduncle or in DRPLA [60]. In contrast, PET imaging and other advanced modalities have been less frequently employed, typically involving smaller cohorts and limited longitudinal data, whereas DAT-SPECT abnormalities may emerge in some of them with disease progression [61,62]. Figure 2 illustrates the characteristic structural MRI findings for each disorder, and Table 3 summarizes the principal neuroimaging biomarker results.

**SCA1:** In the EUROSCA cohort (526 patients; 117 with SCA1), MRI volumetry performed in 48 available SCA1 cases (1.5T MRI) confirmed the classic pattern of olivopontocerebellar atrophy. Moreover, brainstem atrophy correlated with SARA scores as well as with posture and gait impairment [8]. At the same time, spinal cord is also affected, the degree of atrophy correlating with disease progression [43,63]. In presymptomatic SCA1, DTI has detected reduced fractional anisotropy in the dentate nucleus and cerebellar peduncles despite normal MRI, revealing early microstructural degeneration [64].

**SCA2:** The ENIGMA-Ataxia study revealed that white-matter atrophy in cerebellar peduncles and pons is the earliest and strongest biomarker, present even in presymptomatic carriers. Atrophy progresses hierarchically: first involving cerebellar white matter and pons, then cerebellar gray matter (lobules I–IV, IX), spreading to cerebral white matter tracts and finally to striatum, thalamus and cortex [65]. Such changes are detectable even in presymptomatic carriers [66]. In symptomatic patients, cerebellar atrophy in anterior (V) and posterior lobules (VI–IX, Crus I/II) correlates with visuospatial, memory, and executive deficits, linking regional atrophy to domain-specific cognitive impairment [53]. Over one year, imaging studies showed the fastest atrophy progression in the pons and lobule Crus I [67].

**SCA3 (Machado–Joseph disease):** MRI studies revealed reduced corpus callosum, ventricular enlargement, and atrophy of motor (I–VI, VIII) and cognitive (crus) cerebellar lobules, progressing with disease severity [68]. Clinical correlations linked brainstem and cerebellar hemispheric atrophy with posture, gait, and limb kinetic deficits, suggesting that imaging can be a reliable biomarker [8]. SCA3 is not just a cerebellar disorder, as imaging shows widespread cortical atrophy [69], disrupted thalamocortical white matter tracts strongly linked to ataxia severity [70], and altered cerebello-cortical network organization (without faster cerebellar atrophy in larger repeats [71]), establishing these supratentorial changes as key imaging biomarkers. Interestingly, even if the natural history of the disease starts subtentorially (cerebellum, especially lobule X, and substantia nigra) to spread later to cortical regions, the easiest places to measure disease progression are cerebellar peduncles and spinal cord [72,73,74]. Dystonia is a frequent complication, associated with cortical (pre/paracentral, hippocampal) and subcortical (thalami, diencephalon, cerebellar white matter) atrophy, while non-dystonic cases mainly show occipital thinning [75]. 18-FDG-PET hypometabolism was reported in the same regions [76].

In the large ESMI cohort, which included 291 SCA3 carriers, all analyzed MRI volumes and diffusion measures worsened over time, although at different rates and with distinct trajectories. The earliest MRI abnormality was medulla oblongata volume loss, which became abnormal 4.7 years before the estimated onset [77]. Fractional anisotropy (FA) of the inferior cerebellar peduncle (ICP) also declined early, followed by pons volume (−0.6 years), confirming that brainstem degeneration precedes clinical manifestation. Other MRI parameters became abnormal 0.3–14.4 years after onset, following a clear temporal sequence: radial diffusivity (RD) of the ICP, cerebral white matter (CWM) volume, midbrain volume, superior cerebellar peduncle (SCP) volume, and FA of the SCP [77]. In contrast, cortical gray matter (CGM) volume did not decrease beyond two standard deviations below the mean of healthy controls throughout the disease course, indicating relative cortical preservation in comparison with infratentorial and white matter structures [77]. These longitudinal data reveal a hierarchical and region-specific pattern of neurodegeneration, with early and progressive involvement of the medulla, pons, and cerebellar peduncles, supporting MRI-based measures as sensitive and stage-dependent biomarkers of disease progression in SCA3 [77].

**SCA6:** MRI changes in SCA6 are characterized by selective cerebellar cortical atrophy, with mild correlations between cerebellar volume loss and gait impairment, and marked oculomotor abnormalities—including broken pursuit, gaze-evoked nystagmus, and downbeat nystagmus—highlighting the relative sparing of the brainstem. However, in SCA6, brainstem atrophy was found to correlate with disease progression and symptom severity [78]. Morphometric analyses further demonstrated that atrophy in medial superior regions (lobule VI, Crus I, dentate) correlated with poorer motor performance, whereas inferior regions (lobules VIIb–VIIIa) were associated with deficits in executive function [79]. Finally, a case study using ^11^C-ITMM PET in a single patient revealed markedly reduced mGluR1 availability in the vermis and flocculus, suggesting Purkinje cell dysfunction as a sensitive imaging biomarker for research purposes [80].

**SCA7:** Typically combines cerebellar/pontine atrophy with occipital cortical involvement; in a 1-year longitudinal study of SCA7 patients, imaging revealed progressive pontine and medulla atrophy with preserved cerebellum in initial stages, and progressive retinal ONL thinning correlating with severity [81]. In SCA1, 2, 3, and 7, magnetic resonance spectroscopy shows reduced neuronal markers, elevated glial and energetic metabolites, correlating with clinical severity but not genetics, highlighting dynamic metabolic biomarkers of progression [82].

**SCA17:** Although no large longitudinal studies of SCA17 are available, existing reports consistently describe diffuse cortical–subcortical, striatal, and cerebellar atrophy, including a characteristic putaminal rim [48], often with frontostriatal involvement that parallels cognitive and behavioral symptoms [83,84,85]. FDG-PET classically shows hypo metabolism in cerebellum, striatum, and cortical regions [86], although atypical presentations with thalamic and cortical hypometabolism but preserved cerebellar metabolism have also been reported [85].

**DRPLA:** In the Chinese OSCAAR cohort, MRI analyses revealed widespread gray matter loss in symptomatic patients, involving the frontal, parietal, temporal, and occipital cortices, as well as significant volume reduction in subcortical structures, brainstem, cerebellum, and cerebral white matter. In contrast, prodromal individuals exhibited atrophy predominantly confined to the bilateral cerebellar hemispheres, indicating early and regionally selective involvement [87,88]. DTI demonstrated generalized microstructural white matter alterations closely correlated with clinical severity [68]. The progression of brain atrophy is multisystemic, increasing with disease duration and clinical stage, and shows a strong association with both cognitive decline and ataxia severity. Moreover, there is a direct relationship between CAG repeat length and the degree of cerebral and cerebellar atrophy—larger expansions are associated with greater volume loss in cerebellar and brainstem structures, earlier disease onset, and faster clinical progression [87,88]. One descriptive study showed that quantitative susceptibility mapping can detect increased signal in dentate and red nuclei, reflecting iron deposition and possibly neuroinflammation, while conventional MRI revealed mild cerebellar atrophy and white matter hyperintensities [89]. Another case report showed differential MRI finding between late-onset DRPLA, with cerebellar, pontine, and midbrain atrophy with T2 hyperintensities indicating prominent myelin loss, and juvenile-onset, where severe cortical with later white matter involvement were observed [90].

**SCA27B:** In spinocerebellar ataxia type 27B (SCA27B), brain MRI may show mild to moderate cerebellar atrophy, predominantly involving the vermis and superior cerebellar peduncles, although extracerebellar involvement is minimal. Hemispheric atrophy, focal cerebellar gliosis, and nonspecific supratentorial white matter changes have been occasionally reported [91,92,93,94]. The disease course is slowly progressive, with an estimated annual increase of 0.3–0.4 SARA points, and cerebellar atrophy advances gradually without reaching severe stages, even in long-standing disease. However, the relationship between MRI findings and clinical severity is weak or absent, and up to two-thirds of patients may show normal MRI scans despite mild ataxic symptoms. Consequently, conventional MRI has low sensitivity for detecting progression or correlating with clinical severity, Oculovestibular and cognitive symptoms may also occur in the absence of clear MRI abnormalities, highlighting the need for larger longitudinal imaging studies to better define the structural and functional correlates of disease progression in SCA27B [91,92,93,94]. FDG-PET showed hypometabolism in premotor cortex and cerebellar central lobule, supporting motor circuit dysfunction [91,95,96].

**Friedreich ataxia:** In FRDA, neuroimaging abnormalities reflect a combination of early developmental hypoplasia—particularly of cerebellar and brainstem structures—and superimposed progressive neurodegeneration. The largest multicenter, multimodal neuroimaging study to date in Friedreich ataxia TRACK-FA [97] (cross-sectional cohort, 169 patients and 95 controls) demonstrated robust structural and microstructural abnormalities. Compared with controls, patients showed reduced cerebellar volume, atrophy of the superior cerebellar peduncles and dentate nuclei with increased susceptibility and reduced spinal cord cross-sectional area [97]. Diffusion imaging revealed decreased white matter integrity in the superior cerebellar peduncles and cervical cord, while spectroscopy showed lower neuronal-to-glial metabolite ratios. These abnormalities were evident from childhood, reflecting early hypoplasia followed by progressive degeneration, and correlated strongly with disease severity. Notably, dentate nucleus susceptibility increased with advancing stage, whereas volume loss plateaued early [97]. These findings extend those of a diffusion MRI tractography study in 45 FRDA patients, which demonstrated abnormalities along the dentato–thalamo–cortical tract. Damage was most pronounced between the dentate nuclei and red nuclei and near the thalamus, whereas thalamo–cortical segments were relatively preserved. Changes were symmetric and correlated with GAA repeat length and clinical ataxia severity, consistent with a gradient of anterograde degeneration [98]. An interventional DTI study using erythropoietin therapy reported widespread supratentorial increases in white matter anisotropy, without significant cerebellar or brainstem changes [99]. Advanced imaging in FRDA has further revealed selective white matter atrophy of cerebellar tracts linked to clinical severity [100], dentate nucleus volume loss with progressive T2 alterations [101], and combined cortical and nuclear dysfunction [102]. Functional MRI demonstrated compensatory cortical hyperactivation and reorganization [103,104], as well as impaired cerebello-cortical connectivity [105].

***RFC1*-related ataxia (CANVAS):** Early imaging work in CANVAS came from a retrospective review of 18 Australian and New Zealand patients, which demonstrated cerebellar atrophy (89% of patients), especially in the anterior and dorsal vermis (lobules VI–VIIa/b, also known as crus I), with sparing of brainstem and inferior vermis [106]. These observations were later refined into the proposed diagnostic criteria [107], which emphasized that MRI may be normal initially but consistently evolves to show vermal and crus I atrophy with preserved brainstem [106]. The first Japanese case report confirmed this pattern, extending the phenotype with SPECT study, revealing hypoperfusion of cerebellar hemispheres and vermis [108]. More recently, a case series of 19 cases [109] extended the phenotype using molecular imaging: FDG-PET consistently demonstrated cerebellar hypometabolism with preserved striatum and brainstem, distinguishing CANVAS from MSA-C and SCA3; occasional insular–opercular hypometabolism may underlie autonomic features. By contrast, DaT-SPECT proved unreliable due to variability, and cardiac MIBG scintigraphy remains inconclusive, overlapping with SCA3 [109].

**SPG7**: Neuroimaging studies in spastic paraplegia type 7 (SPG7) consistently reveal combined corticospinal and cerebellar system involvement. An MRI study in 11 genetically confirmed patients showed predominant cerebellar atrophy, especially of the superior vermis and hemispheres, often accompanied by brainstem and spinal cord thinning, while cerebral cortex and white matter were largely preserved, supporting the interpretation of SPG7 as a spastic-ataxic syndrome, not a purely pyramidal disorder [110]. Advanced multimodal MRI extended this view by studying 31 hereditary spastic paraplegia patients (including SPG7) with voxel-based morphometry, spectroscopy and diffusion tensor imaging. Bilateral thalamic and right prefrontal cortex gray matter loss was found, along with widespread white-matter microstructural abnormalities involving corticospinal, callosal, and associative tracts, while the primary motor cortex remained relatively spared. Metabolically, reduction in N-acetylaspartate and increased choline and myo-inositol contents suggested axonal dysfunction and gliosis. Unexpectedly, longitudinal DTI over 2.5 years showed increased fractional anisotropy and decreased diffusivity, pointing to possible microstructural reorganization or compensatory remyelination rather than progressive degeneration [111].

**FXTAS**: Radiological findings in FXTAS are well defined, with MRI hallmarks including middle cerebellar peduncle (MCP) hyperintensities and, less consistently, corpus callosum splenium lesions [112]. The MCP sign is more predictive than splenial changes [113], and it can appear even in premutation carriers without clinical disease [114]. White matter hyperintensities, especially in frontal periventricular regions, are also described [115]. Advanced MRI (DTI, MRS) supports a model of widespread white matter degeneration: reduced fractional anisotropy and altered diffusivity in the MCP, superior peduncles, fornix, and callosal fibers correlate with executive dysfunction, while lower MCP NAA/Cr and Ch/Cr ratios predict reduced fluency, suggesting axonal and myelin compromise [116,117]. Clinically, MRI correlates with varied presentations: ataxia-dominant phenotypes [118], dementia of frontal profiles [119], sometimes accelerated by Alzheimer co-pathology [120], and rare parkinsonism linked to nigrostriatal degeneration [121]. Female pre-FXTAS patients have distinctive splenial alterations [122].

**Table 3:** Principal neuroimaging biomarker results.

Disorder	MRI Key Findings (Figure 2) Advanced MRI (DTI/7T/MRS)	Nuclear Medicine PET SPECT
**SCA1** [43,63,64,82,123,124,125]	Olivopontocerebellar atrophy. Spinal involvement.	Hypometabolism in the cerebellum and brainstem.
	Reduced FA in cerebellar peduncles. Spectrometry revealed reduced neuronal markers, elevated glial and energetic metabolites.	DAT-SPECT altered/normal.
**SCA2** [66,82,124,125,126]	Olivopontocerebellar atrophy, later involvement of subcortical structures and cortex.	Hypometabolism in the cerebellar cortex, dentate nucleus, brainstem (particularly the pons), and parahippocampal cortex.
	Spectrometry revealed reduced neuronal markers, elevated glial and energetic metabolites.	DAT-SPECT may be altered with/without parkinsonian phenotype.
**SCA3 (MJD)** [68,69,70,71,72,73,74,76,77,82,124]	Olivopontocerebellar atrophy: Progressive pons, cerebellar, superior cerebellar peduncles and spinal atrophy, frequent involvement of substantia nigra and progression to basal ganglia and cortical area.	Hypometabolism in the cerebellar cortex, basal ganglia–putamen. Cortical (motor/premotor) and thalamic hypometabolism in dystonic cases.
	Early reduction in FA in inferior peduncle and white matter preceding gray matter loss. Spectrometry with reduced neuronal markers, elevated glial and energetic metabolites.	Altered DAT-SPECT parkinsonian phenotype dopa-responsive.
**SCA6** [78,79,80,124,127]	Selective cerebellar atrophy.	Cerebellar hypometabolism. Marked reduction in mGluR1 availability in vermis and flocculus.
	Microstructural alterations in the cerebellar white matter and cerebellar peduncles.	Normal DAT-SPECT.
**SCA7** [81,82,128]	Olivopontocerebellar atrophy, medullar and occipital atrophy.	Marked hypometabolism in the cerebellum and brainstem.
	Spectrometry revealed reduced neuronal markers, elevated glial and energetic metabolites.	Limited data
**SCA17** [48,83,84,85,86,129]	Cerebellar atrophy, frontostriatal (caudate and putamen) atrophy, putaminal rim sign.	Hypometabolism in cerebellum, striatum and cortex, atypical presentation with thalamocortical hypometabolism and preserved cerebellar metabolism have been described.
	Limited data.	DAT-SPECT altered in advances stages.
**SCA27B** [91,95,96]	Mild cerebellar atrophy predominantly affecting the superior vermis/Normal.	Hypometabolism in premotor cortex and cerebellar central lobule.
	Microstructural alterations in the superior cerebellar peduncle, with reduced FA and increased MD.	Limited data.
**DRPLA** [87,88,89,90,130]	Widespread cortical, cerebellar, pontine and midbrain atrophy, white matter hyperintensities, and pontine hypointensity	Striatal hypometabolism.
	Generalized white matter microstructural alterations correlating with clinical progression.	Limited data.
**FRDA** [56,97,99,100,101,103,104,105,131]	Mild cerebellar atrophy, predominant in dentate and superior peduncle, reduced cervical spinal cord cross-section.	Cerebellar hypometabolism.
	Iron deposition in the dentate nucleus with susceptibility-weighted signal changes Superior peduncle and cervical cord degeneration in DTI, fMRI shows compensatory cortical hyperactivation and reorganization.	Limited data.
***RFC1*** **(CANVAS)** [106,107,108,109,132,133,134]	Cerebellar vermian atrophy, mainly anterodorsal.	Mild hypoperfusion of cerebellar hemispheres and vermis, preserved striatal metabolism.
	DTI changes mainly involve dorsal sensory tracts, deep white matter, corpus callosum, and major cerebral tracts.	DAT-SPECT can be altered (even without parkinsonism).
**SPG7** [110,111]	Cerebellar atrophy, most prominently cerebellar vermis, hyperintensity of dentate nucleus.	Limited data.
	DTI: white matter changes characterized by reduced FA and increased MD in the frontal lobes, corticospinal tracts, and brainstem.	Limited data.
**FXTAS** [112,113,114,115,116,117,135,136]	Hyperintensity of the middle cerebellar peduncle - MCP sign, diffuse WM hyperintensity.	Hypometabolism in the frontotemporal and cerebellar regions.
	DTI with widespread WM damage; MRS abnormalities suggesting myelin damage (low NAA/Cr and Ch/Cr ratios).	Significant reduction in DAT uptake in the bilateral putamen, with or without parkinsonism.

Ch, choline; Cr, creatine; DAT-SPECT, dopamine transporter single-photon emission computed tomography; DTI, diffusion tensor imaging; FA, fractional anisotropy; FDG-PET, fluorodeoxyglucose positron emission tomography; fMRI, functional magnetic resonance imaging; MCP, middle cerebellar peduncle; MD, mean diffusivity; MRS, magnetic resonance spectroscopy; mGluR1, metabotropic glutamate receptor type 1; NAA, N-acetylaspartate; PET, positron emission tomography; SPECT, single-photon emission computed tomography; WM, white matter.

### 3.2. Fluid Biomarkers

Within the spectrum of fluid biomarkers in cerebellar ataxias, several categories can be distinguished according to the underlying biological process. The first group includes disease-specific biomarkers directly linked to the primary molecular defect, such as mutant or total ataxin-3 in SCA3 and frataxin in Friedreich’s ataxia, which reflect the pathogenic protein burden and serve as indicators of target engagement and therapeutic efficacy in gene- or protein-directed interventions. The second group encompasses biomarkers of neurodegeneration, notably neurofilament light chain (NfL) and tau proteins, which signal axonal injury and neuronal loss and generally correlate with disease severity and progression across subtypes. Finally, metabolic and glial biomarkers—including markers of oxidative stress, mitochondrial dysfunction, lipid metabolism, and astrocytic activation (e.g., GFAP)—are emerging as complementary indicators of secondary pathophysiological processes. Collectively, these biomarkers offer an integrated framework to understand disease mechanisms, monitor neurodegeneration, and evaluate treatment response in cerebellar ataxias (Table 4).

Among them, NfL has emerged as the most robust and reproducible biomarker of neuroaxonal damage across inherited ataxias. Elevated serum or plasma NfL levels have been consistently reported in preataxic and manifest carriers of SCA1, SCA2, SCA3, and SCA7, showing a stepwise increase from presymptomatic to symptomatic stages and strong correlations with clinical severity, supporting its use for disease staging and as a secondary outcome in interventional trials [49,91]. However, not all ataxias exhibit NfL elevation. In SCA6 and SCA27B, NfL concentrations are comparable to controls, likely reflecting their slowly progressive, predominantly cerebellar pathology with limited extracerebellar axonal loss. These “atypical” trajectories suggest that in very slowly progressive or late-onset ataxias, the rate of axonal injury may be too low to produce measurable differences. Conversely, in Friedreich’s ataxia, NfL levels are high early in life but decline over time despite clinical worsening, indicating a biphasic neurodegenerative process or late-stage “burnout” phenomenon.

Although NfL has been primarily studied in blood, several studies have also examined its CSF concentrations, showing a strong correlation between CSF and plasma levels and confirming that peripheral measurements reliably reflect central neuroaxonal injury [137,138,139]. This cross-matrix consistency reinforces the translational value of NfL as a minimally invasive biomarker suitable for longitudinal monitoring and clinical trials.

While NfL is now established as a reliable but non-specific marker of neuronal injury, data remain scarce for less frequent genetic ataxias, where natural history and biomarker trajectories are poorly defined due to small and fragmented cohorts [91].

Beyond its prognostic role, serum NfL also holds diagnostic utility, distinguishing degenerative ataxias from controls. In multiple system atrophy of the cerebellar type (MSA-C), NfL levels are markedly elevated owing to widespread axonal degeneration in long tracts such as the spinocerebellar and corticospinal pathways. NfL differentiates MSA-C from sporadic adult-onset ataxia (SAOA) with good accuracy (AUC = 0.74, 95% CI 0.59–0.89) and separates SCA patients from healthy controls with high precision (AUC = 0.91, 95% CI 0.81–1.00) [91].

**SCA1:** In *ATXN1* expansion carriers, mean plasma NfL levels were 24.8 ± 11.1 pg/mL, significantly higher than in controls (10.2 ± 4.5 pg/mL), with an optimal diagnostic cut-off of 16.9 pg/mL. NfL levels correlated with CAG repeat size and increased in parallel with clinical worsening over two years (SARA 10.7 ± 6.2 → 13.1 ± 7.0). Despite longitudinal stability, higher baseline NfL values were associated with faster clinical progression. One premanifest carrier with near-threshold NfL (13.3 pg/mL) converted to symptomatic disease, supporting NfL as a sensitive early biomarker in this genotype [140].

**SCA2:** *ATXN2* expansion carriers showed mean NfL concentrations of 20.3 ± 6.3 pg/mL (cut-off 19.1 pg/mL), significantly higher than in controls. Baseline NfL values correlated with disease severity (SARA and CCFS) and predicted subsequent cerebellar volume loss (β = −1.0 × 10^−4^ ± 3.7 × 10^−5^), confirming its strong prognostic value. Longitudinally, NfL levels remained stable (20.3 pg/mL at baseline; 20.1 pg/mL at follow-up) despite measurable clinical progression (SARA + 2.1 points) [140].

A TR-FRET immunoassay was developed to quantify soluble polyQ-expanded ataxin-2 (ATXN2) with high specificity, validated in mouse tissue and patient-derived fibroblasts and iPSC-derived cortical neurons. The assay detected small expression changes (down-regulation with ATXN2 siRNA; up-regulation with starvation) but serum levels fell below the assay’s limit of quantification, indicating that ultrasensitive platforms (e.g., Simoa or SMC) and CSF analysis will likely be required for clinical application. Overall, soluble polyQ-ATXN2 represents a promising pharmacodynamic and target-engagement biomarker for SCA2, complementary to degeneration markers such as NfL and tau, pending longitudinal validation [141].

**SCA3:** Across studies, ATXN3 expansion carriers consistently exhibit the highest plasma and CSF NfL concentrations among all polyglutamine SCAs, confirming its value as a robust biomarker of neuroaxonal damage and disease staging. In the Paris Brain Institute cohort, mean plasma NfL reached 34.9 ± 10.5 pg/mL, significantly exceeding levels observed in SCA1 and SCA2, with a diagnostic cut-off of 16.0 pg/mL (AUC = 0.99). NfL correlated strongly with disease severity (SARA r = 0.76; CCFS r = 0.59) and duration (r = 0.57), and inversely with pons volume (r = −0.71). Although overall NfL levels remained stable at follow-up (33.1 ± 9.4 pg/mL), one premanifest carrier with near-threshold NfL (15.9 pg/mL) converted to symptomatic disease within two years, supporting its utility in detecting preclinical neurodegeneration [140]. Consistently, in the Chinese SCA3 cohort (133 ATXN3 carriers and 100 controls), serum and CSF NfL levels were markedly elevated compared with controls (CSF: 4262 ± 1762 vs. 472 ± 210 pg/mL; serum: 41.5 ± 14.8 vs. 9.1 ± 3.3 pg/mL), showing a strong serum–CSF correlation (r = 0.92). Elevated NfL was evident even in preclinical carriers (15.0 ± 7.5 pg/mL) versus controls (6.9 ± 2.7 pg/mL) and further increased in manifest disease (37.6 ± 13.5 pg/mL). ROC analyses demonstrated high diagnostic accuracy (AUC = 0.98 for manifest; 0.84 for preclinical), with optimal thresholds of 20 pg/mL and 10 pg/mL, respectively. NfL correlated positively with SARA (r = 0.55) and ICARS (r = 0.55) scores, and inversely with cerebellar (r = −0.42) and brainstem volumes (r = −0.43), independently of age and CAG repeat length. Notably, NfL increased in late-preclinical carriers, preceding overt ataxia and pontocerebellar atrophy [137]. Plasma total tau (t-tau) levels are elevated in SCA3 mutation carriers, particularly in individuals under 50 years of age, compared to controls. Importantly, cerebrospinal fluid (CSF) concentrations of t-tau and phosphorylated tau (p-tau181) are higher in preataxic carriers than in symptomatic patients, suggesting tau may reflect neuronal injury in the prodromal phase of SCA3 and could be useful for early disease detection or staging [142].

Expanded polyQ ATXN3 is readily detectable and markedly elevated in both CSF and plasma, distinguishing patients from controls with excellent diagnostic precision (AUC ≈ 1.00). CSF polyQ ATXN3 further differentiates symptomatic from presymptomatic carriers (AUC ≈ 0.89) and decreases after ATXN3 siRNA treatment, validating it as a pharmacodynamic biomarker of target engagement in clinical trials. Overall, polyQ ATXN3 represents a diagnostic and treatment-response biomarker in SCA3, complementary to NfL [143].

**SCA6:** In contrast to other polyglutamine SCAs, plasma NfL levels are not significantly elevated in SCA6 compared with healthy controls. In the meta-analysis by [49], which pooled data from over 1000 hereditary ataxia patients, SCA6 was the only subtype without a significant increase in plasma or serum NfL. Mean values were approximately 2.6 log pg/mL in SCA6 and 2.5 log pg/mL in controls (*p* > 0.05), indicating minimal difference. This likely reflects the slowly progressive and primarily cerebellar pathology of SCA6 [144].

**SCA7:** In SCA7, both NfL and GFAP are elevated, reflecting active neurodegeneration. Plasma NfL averaged 21.6 pg/mL versus 8.2 pg/mL in controls, and CSF NfL 2615 versus 415 pg/mL. NfL correlated strongly with CAG repeat length (r = 0.93) and with clinical worsening over two years (β = 0.007 ± 0.003), whereas GFAP elevation appeared less specific. Carriers with NfL ≥16 pg/mL often displayed subclinical neurological findings, while those <10 pg/mL remained asymptomatic, indicating NfL’s potential to detect early neurodegeneration and predict phenoconversion [140,145].

**DRPLA:** In the OSCAAR cohort, plasma NfL was 38.2 pg/mL in manifest patients, 13.7 pg/mL in prodromal carriers, and 9.8 pg/mL in controls, showing a clear stepwise increase with advancing disease severity. This gradient mirrors the transition from localized cerebellar involvement in the prodromal stage to widespread neurodegeneration in the manifest phase [88].

**SCA27B:** Wilke C et al. reported that plasma NfL concentrations averaged 22.7 ± 8.2 pg/mL in SCA27B patients, comparable to age-matched healthy controls (median age 60 years) and markedly lower than in faster-progressing SCAs. These results indicate that NfL is not elevated in SCA27B, reflecting the mild and slowly progressive neurodegeneration typical of this subtype. Consequently, NfL may lack sensitivity for monitoring disease progression in SCA27B. Larger longitudinal studies are required to determine its diagnostic and prognostic value [146].

**Friedreich’s Ataxia (FRDA):** Across two large international cohorts (EFACTS and FACOMS), plasma NfL concentrations averaged 37.4 ± 19.7 pg/mL in FRDA patients compared with 15.2 ± 6.4 pg/mL in healthy controls [147]. Diagnostic accuracy was excellent in individuals younger than 20 years (AUC = 1.00), high up to age 50 (AUC ≈ 0.89), and declined thereafter, approaching control values beyond 40 years. Longitudinally, NfL declined by approximately 13% per year in pediatric patients (<18 years) and by 7–10% per year in young adults (18–35 years), reflecting a natural downward trend in untreated individuals but remaining higher than controls at all time points. NfL correlated inversely with GAA1 repeat length, but not consistently with SARA or mFARS scores [147].

**Table 4:** Laboratory biomarkers.

Disease	Biomarker	Key Findings
**SCA1** [140]	NfL	Increased in preataxic and ataxic carriers (plasma ≈ 24.8 ± 11.1 pg/mL vs. 10.2 ± 4.5 pg/mL in controls); correlates with CAG repeat length and clinical progression.
**SCA2** [140,141]	NfL	Elevated in preataxic and ataxic carriers (plasma ≈ 20.3 ± 6.3 pg/mL, cut-off 19.1 pg/mL); baseline levels predict cerebellar volume loss. Stable longitudinally despite clinical worsening.
	PolyQ-ATXN2	Target engagement marker. Requires further validation in patient cohorts
**SCA3** [137,140,142,143]	NfL	Significantly elevated in plasma and CSF (plasma ≈ 41.5 ± 14.8 pg/mL vs. 9.1 ± 3.3 pg/mL; CSF ≈ 4262 ± 1762 pg/mL vs. 472 ± 210 pg/mL); correlates with SARA, ICARS, and cerebellar atrophy; strong serum–CSF correlation (r ≈ 0.9). (Higher levels across PolyQ ataxia)
	PolyQ-ATXN3	Target engagement marker. Validated in plasma & CSF
	Tau	Elevated in preataxic >> ataxic patients >> controls
	GFAP	Increased levels no correlation with disease progression
**SCA6** [49,144]	NfL	No significant differences vs. controls (plasma ≈ 2.6 log pg/mL vs. 2.5 log pg/mL)
**SCA7** [140,145]	NfL	Increased levels in plasma (≈21.6 pg/mL vs. 8.2 pg/mL) and CSF (≈2615 pg/mL vs. 415 pg/mL); strongly correlates with CAG repeat length and clinical progression.
	GFAP	Elevated but less specific than NfL; potential marker of astroglial activation.
**SCA17**	Limited data	Further studies are needed
**DRPLA** [88]	NfL	Stepwise increase in plasma: controls (≈9.8 pg/mL) < preataxic (≈13.7 pg/mL) < ataxic (≈38.2 pg/mL); correlates with disease severity and CAG repeat length.
**SCA27B** [146]	NfL	Comparable to age-matched controls (≈22.7 ± 8.2 pg/mL); not significantly elevated; limited sensitivity for progression.
**FRDA** [147,148]	NfL	Elevated and correlated with severity (plasma ≈ 37.4 ± 19.7 pg/mL vs. 15.2 ± 6.4 pg/mL in controls). Levels decline with disease progression
	Frataxin protein	Target-engagement marker; lower levels correlate with GAA expansion and clinical severity. Stable longitudinally.
	Tau	Increased in younger patients
	GFAP	Increased in younger patients
	Metabolic	Mitochondrial/oxidative alterations in G-CSF and antioxidant trials
***RFC1*** **(CANVAS)** [149]	NfL	Increased serum levels (≈24.3 ± 9.2 pg/mL vs. 12.4 ± 4.6 pg/mL); higher in patients with cerebellar involvement (≈28 pg/mL); independent of disease duration.
**SPG7**	Limited data	Further studies are needed
**FXTAS**	Limited data	Further studies are needed

Note: Quantitative NfL values are derived from different studies using heterogeneous assays, sample sizes, and disease-stage compositions. Therefore, inter-study comparisons should be interpreted as approximations rather than direct equivalences. Variability across analytical platforms, kits, cohort demographics, and disease severity can influence absolute concentrations (although the analytical CV of NfL assays is typically <10%). CAG, cytosine–adenine–guanine (trinucleotide repeat); CSF, cerebrospinal fluid; GAA, guanine–adenine–adenine (trinucleotide repeat); G-CSF, granulocyte colony-stimulating factor; GFAP, glial fibrillary acidic protein; NfL, neurofilament light chain; polyQ, polyglutamine.

Tau (t-tau) levels were moderately elevated across all ages, while GFAP appeared increased only in younger patients (<13 years), and UCH-L1 exhibited high analytical variability and poor reproducibility. Collectively, these findings indicate that NfL is a sensitive marker of early neuroaxonal injury in FRDA and may serve as a pharmacodynamic biomarker in clinical trials. Tau and GFAP could provide complementary insight into glial or compensatory mechanisms, though further longitudinal and mechanistic studies are needed to clarify their clinical relevance [147].

Frataxin as a disease and pharmacodynamic biomarker: Lynch et al. quantified both mitochondrial (FXN-M) and erythroid (FXN-E) isoforms of frataxin using a triple quadrupole LC–MS assay, achieving sensitive and reproducible quantification of frataxin deficiency. Both isoforms correlated strongly with GAA1 repeat length and, to a lesser extent, with age [148]. FXN-E and FXN-M correlated with clinical severity scores (mFARS, FDS), with FXN-E showing stronger associations due to lower biological variability from its erythroid origin [148]. Frataxin levels remained stable longitudinally, supporting their use for serial monitoring in clinical trials [148].

***RFC1*-related ataxia (CANVAS):** In patients with biallelic *RFC1* AAGGG repeat expansions, serum NfL concentrations were significantly elevated compared with healthy controls, reflecting axonal degeneration within both the peripheral and central nervous systems. In the largest multicenter study to date (n = 61 patients, 48 controls), mean serum NfL was 24.3 ± 9.2 pg/mL in *RFC1* patients versus 12.4 ± 4.6 pg/mL in age- and sex-matched controls (AUC = 0.93) [149]. NfL correlated moderately with age and remained significantly elevated across all clinical subtypes, including isolated sensory neuropathy. Patients with cerebellar involvement exhibited higher NfL (27.9 ± 9.6 pg/mL) compared with those without cerebellar dysfunction (21.8 ± 7.0 pg/mL), an association that persisted after adjusting for age and sex (β = 0.26, *p* = 0.034). No correlation was found with disease duration or ambulation, suggesting that NfL increases early and plateaus thereafter. Longitudinal studies are needed to confirm its prognostic significance [149].

#### Metabolic Markers

Although metabolic biomarkers have not been as extensively investigated in large longitudinal cohorts as neurodegenerative markers such as NfL, emerging evidence suggests that they may also provide valuable insights into disease mechanisms and therapeutic monitoring. Metabolic profiling has become increasingly relevant across hereditary ataxias, identifying disease-specific biochemical fingerprints. In SCA7, plasma acylcarnitine and amino acid imbalances have been demonstrated, suggesting mitochondrial energy disruption [46]. Similarly, SCA2 patients enrolled in a randomized zinc sulfate trial showed measurable shifts in oxidative and antioxidant metabolic balance [150]. These results highlight that metabolic markers may function not only as mechanistic readouts but also as targets for nutritional or metabolic interventions. In FXTAS, metabolomic studies have been particularly informative. Allopregnanolone treatment improved plasma GABA-related metabolite profiles [151], directly linking therapeutic modulation to measurable biochemical change. Larger untargeted metabolomic analyses confirmed broad disturbances in lipid and energy metabolism, which correlated with clinical severity [152]. For Friedreich’s ataxia (FRDA), while frataxin remains the central biomarker, additional metabolic parameters—such as mitochondrial respiratory intermediates and redox balance—have been explored in erythropoietin and EPO and antioxidant trials [153,154]. The integration of metabolomic outcomes into both natural history and interventional studies is increasingly recognized to capture the global metabolic burden of FRDA beyond protein deficiency alone.

### 3.3. Digital/Neurophysiological Biomarkers

#### 3.3.1. Digital and Gait/Postural Control Biomarkers (Table 5)

Recent years have seen the emergence of digital gait and postural control assessments as promising biomarkers in hereditary ataxias. Using wearable inertial sensors and instrumented posturography, these approaches provide objective and ecologically valid measures that may overcome the limited sensitivity of conventional clinical scales. In SCAs, sensors placed on the wrist, ankle, pelvis, and trunk have been employed to quantify gait variability, postural stability, and motor coordination. These devices can discriminate patients from healthy controls, estimate ataxia severity, and monitor disease progression through machine-learning algorithms and submovement analysis [155,156,157,158,159].

The most relevant parameters include stride length and duration variability, double-support time, foot-out angle, trunk acceleration variability, axial movement entropy, and kinematics of specific motor tasks such as the finger-to-nose and diadochokinesis tests [155,156,157,159]. Furthermore, several studies have explored the use of wearable sensors to evaluate fall risk and quantify responses to rehabilitation interventions, including vibrotactile feedback training [160].

Genotype-specific studies refine these concepts and support the trial-readiness of digital gait endpoints. In a multicenter wearable-sensor study including **SCA1**, a standardized 2 min overground walk with six synchronized inertial sensors (feet, wrists, sternum, and lumbar) showed that step-to-step variability metrics (variability of double-support time, foot-progression angles, and foot clearance during mid-swing) provided the strongest discrimination from controls and tracked severity, with some prodromal carriers already exhibiting abnormal variability signals [157]. In early-stage **SCA2**, a similar 2 min instrumented walk identified lateral step deviation and composite spatial variability indices as the most informative features: these metrics differentiated SCA2 from controls, correlated with SARA and genetically estimated time to onset, and progressed significantly over one year even when SARA remained stable, yielding favorable sample-size estimates for future trials [161]. In **SCA3**, both camera-based motion capture and wearable sensors have shown that mediolateral sway and stride-length variability change significantly over 12 months, outperforming clinical scales and reducing required trial sample sizes [162]. Markerless depth-camera systems and Azure-Kinect–based analyses further highlighted increased step width, mediolateral margins of stability, and altered hip/knee/ankle kinematics as practical clinic-feasible screening and monitoring markers [163]. In **SCA6**, instrumented walkway analyses across the disease spectrum (including presymptomatic carriers) demonstrated that gait impairment precedes clinical onset, as presymptomatic individuals already exhibited increased step-width and step-time coefficients of variation, whereas symptomatic patients showed a broader spatiotemporal deficit [164,165]. Marker-free RGB-D camera assessments in degenerative cerebellar ataxia cohorts, including SCA6, have also identified mediolateral truncal sway during walking and arrhythmicity on stepping-in-place as robust digital biomarkers, while real-life wearable recordings indicate that turning-based dynamic-balance metrics detect preataxic change and one-year progression with large within-subject effects [166].

**Friedreich’s ataxia** shows a particularly robust digital gait signature. Across GAITRite and IMU-based studies, adults with FRDA walk markedly slower, with shorter steps and substantially higher stride-length variability than matched controls; this variability relates closely to dynamic balance measures such as the Berg Balance Scale and Limits of Stability [167]. Spatiotemporal metrics correlate with FARS and disease duration, and pediatric kinematic analyses reveal characteristic stance-phase knee and ankle extension patterns that can be more sensitive than clinical scales to short-interval change. Full-body IMU suits combined with machine-learning approaches have predicted future SARA and SCAFI scores several months in advance and cross-sectionally correlate with FXN expression, supporting the prognostic value of sensor-derived biomarkers [168]. Complementary studies show that real-life step counts and mediolateral postural indices exhibit large responsiveness over 6–12 months, informing endpoint selection and sample-size planning for interventional trials [169,170]. Although genotype-specific digital gait studies for **SPG7** remain limited, available quantitative evidence from hereditary spastic paraplegias—of which SPG7 is a frequent cause—demonstrates a consistent spatiotemporal profile characterized by slower gait speed, shorter steps, and increased temporal variability (e.g., step- and stride-time coefficients of variation). These abnormalities reflect the mixed spastic–ataxic gait phenotype typical of SPG7 and align with the broader digital biomarker framework observed across hereditary ataxias, suggesting that IMU-derived variability measures and turning-based dynamic-balance metrics are likely to be informative once systematically explored in SPG7 cohorts [171].

In **FXTAS**, inertial-sensor–instrumented Timed Up and Go paradigms demonstrate a global gait and transition deficit—slower stride velocity and cadence, longer gait cycles, increased double-limb support, higher stride-to-stride variability, and slower, prolonged turns—that discriminates affected premutation carriers from controls while unaffected carriers perform near normal [172]. Several gait and turning measures correlate with the Berg Balance Scale and Functional Independence Measure, supporting their clinical validity. Single-lumbar IMU posturography further reveals larger sway area, higher jerk, and greater RMS sway in FXTAS, with asymptomatic premutation carriers already showing elevated RMS sway under easier stance conditions, suggesting prodromal sway markers for onset detection and trial enrichment [173].

Objective posturography has also demonstrated value in preclinical stages. A four-year prospective study revealed progressive stance instability in asymptomatic SCA1 carriers, supporting postural sway as a sensitive preataxic marker [174]. Complementary approaches, such as wearable proprioceptive stabilizers, have been shown to improve gait stability and may serve both as rehabilitative tools and as quantitative outcome measures [175]. Collectively, these data indicate that sensor-derived metrics of step-to-step variability, dynamic balance, and free-living mobility constitute disease-relevant, scalable digital biomarkers that are increasingly ready for deployment as outcome measures in hereditary ataxia trials.

#### 3.3.2. Oculomotor & Vestibular Biomarkers (Table 5)

Although a comprehensive review of oculomotor alterations across ataxia subtypes exceeds the scope of this article, and the main clinical features by genotype are summarized in Table 5, recent evidence nonetheless clarifies their diagnostic and biomarker potential. A systematic review of quantitative oculomotor testing in hereditary ataxias (89 studies; 1541 patients) concluded that saccadic parameters—particularly latency, peak velocity, and accuracy—are the most consistently informative cross-genotype measures, while other paradigms such as fixation and saccadic intrusions in Friedreich’s ataxia (FRDA), smooth pursuit in SCA17, and quantitative head-impulse testing (qHIT) in SCA3 and SCA6 provide additional genotype-specific insights [176]. Across subtypes, video-oculography reliably discriminates patients from controls and correlates with clinical severity and, when available, imaging markers. Distinct oculomotor “fingerprints” recur by genotype—for example, marked saccadic slowing in SCA2, impaired vestibulo-ocular reflex on qHIT in *RFC1*-related ataxias, downbeat nystagmus in SCA6, and frequent square-wave jerks in FRDA—supporting both diagnostic utility and biological face validity.

**Table 5:** Digital Biomarkers.

Disorder	Digital and Gait/Postural Control Biomarkers	Oculomotor & Vestibular Biomarkers
**SCA1** [174,176,177]	Preataxic: progressive stance instability on posturography; increased without visual cue	GEN, abnormal pursuit, dysmetric and slow saccades
**SCA2** [161,176,177]	Lateral step deviation (wearable sensors during walking) captures 1-year progression in early-stage and preataxic carriers	Marked slow saccade; preserved Quantitative Head Impulse Test (qHIT) gain
**SCA3 (MJD)** [162,176,177]	Stride length variability (CV) and lateral sway detect 1-year progression in early/preataxic	Progressive ophthalmoparesis, hypermetric saccades, GEN, reduced pursuit velocity with saccadic intrusions, moderately vertical saccade velocity & qHIT reduced gain
**SCA6** [165,176,177]	Lateral Velocity Change (LVC) during turning discriminates carriers from controls and detects 1-year progression also in preataxic group	DBN, GEN almost universal; abnormal pursuit; hypermetric saccade. Frequent saccadic intrusion, qHIT markedly reduced gain.
**SCA7** [178]	Limited data	Cone–rod retinal degeneration (SCA7-RD) correlated with ataxia severity and CAG repetition
**SCA17** [176]	Limited data	Abnormal pursuit, slow saccadic movements
**SCA27B** [179]	Limited data	DBN, VOR alteration, GEN
**DRPLA** [180]	Limited data	Nystagmus, dysmetric saccades, supranuclear palsy (advances stages)
**FRDA** [176,181,182,183]	Sway and postural stability measured by posturography correlate strongly with FARS and SARA, particularly under eyes-closed conditions. Gait speed progressively declined and postural instability markedly increased over two years	Oculomotor: frequent saccadic intrusion-SWJ and GEN, horizontal nystagmus, broken pursuit, saccade dysmetria and qHIT strongly reduced gain Optic neuropathy: thinning of pRNFL and macular inner retinal layers on OCT.
***RFC1*** **(CANVAS)** [176,184]	Limited data	DBN frequent, strongly reduced qHIT gain. Absent VOR on head impulse test (“pivotal sign”), bilateral vestibular loss in caloric and vHIT
**SPG7** [185]	Limited data	Progressive retinal atrophy measured by OCT
**FXTAS** [173,186,187]	Larger sway area, increased variability, and greater postural sway jerk	Nystagmus, slow saccades, and fixation abnormalities in advanced stages

DBN, downbeat nystagmus; GEN, gaze-evoked nystagmus; LVC, lateral velocity change; MJD, Machado–Joseph disease; OCT, optical coherence tomography; pRNFL, peripapillary retinal nerve fiber layer; qHIT, quantitative head impulse test; SWJ, square-wave jerks; VOR, vestibulo-ocular reflex.

Recent technological advances have extended oculomotor assessment beyond laboratory paradigms toward ecologically valid, high-frequency digital monitoring. In a multicenter study, Brandon Oubre et al. analyzed 102 individuals with ataxia (36 SCAs, 12 FRDA, and 5 MSA) and 70 healthy controls using binocular eye-tracking at 1000 Hz during a naturalistic reading task. From these data, the Reading Eye Abnormality Digital (READ) score was developed—a composite metric integrating saccadic and fixation dynamics through machine-learning regression. The READ score demonstrated excellent reliability (intraclass correlation coefficient = 0.96) and strong correlations with the Brief Ataxia Rating Scale (BARS; r = 0.82), oculomotor (r = 0.52), and speech (r = 0.73) subscores, as well as patient-reported outcomes (PROM-Ataxia, r = 0.51; Dysarthria Impact Scale, r = 0.53). Importantly, the READ score detected subclinical oculomotor and speech abnormalities (AUC = 0.69 and 0.72, respectively) and captured disease progression over time (d = 0.36), outperforming conventional clinical scales, which did not reach statistical significance (d = 0.27). These findings support digital eye-movement measures as sensitive, objective biomarkers for early detection and longitudinal monitoring in ataxia clinical trials [188].

#### 3.3.3. Neurophysiology & Other Functional Biomarkers

Neurophysiological measures have been explored as early and functional biomarkers in hereditary ataxias. In Machado–Joseph disease, subclinical abnormalities have been documented in presymptomatic carriers, with electrophysiological and non-motor features preceding the onset of ataxia, such as altered vestibular evoked potentials and REM sleep atonia loss [189]. Physiological responsiveness has also been studied through whole-body vibration, which modulated peripheral circulation and neuromuscular activity in patients with hereditary ataxia, suggesting preserved functional pathways that could be leveraged both therapeutically and as outcome measures [190]. In FXTAS, electrophysiology has provided markers of cognitive dysfunction and treatment response. Event-related potentials captured attentional deficits and were modulated by memantine in a randomized trial, supporting their role as objective functional biomarkers [191]. In channelopathies, multimodal evaluation has further expanded the scope of neurophysiology. In a family with a novel CACNA1A mutation, consistent electrophysiological abnormalities were observed despite clinical variability, underscoring its value in defining disease expression [192].

## 4. Conclusions

The landscape of biomarkers in hereditary ataxias is rapidly expanding, mirroring both the biological heterogeneity of these disorders and the clinical need for reliable diagnostic, staging, and pharmacodynamic tools. Across modalities, several strengths have emerged: MRI volumetry and DTI remain the most mature biomarkers, supported by robust longitudinal evidence; fluid biomarkers such as neurofilament light chain (NfL) and frataxin offer sensitive indicators of neuronal injury or mitochondrial dysfunction; and digital gait and oculomotor metrics increasingly provide ecologically valid, quantifiable functional readouts. Collectively, these biomarkers deepen phenotypic characterization and offer measurable endpoints for natural history studies and early-phase clinical trials.

Despite this progress, important limitations remain. Most biomarkers act as indirect proxies rather than validated surrogate endpoints, and their performance varies substantially across genotypes. Imaging protocols, fluid assays, and digital platforms lack harmonization, restricting cross-cohort comparability and generalizability. Biomarker sensitivity differs between early-onset and adult-onset phenotypes, and only a few studies have established clinically meaningful change thresholds—an essential requirement for regulatory qualification. Moreover, evidence is uneven across disorders: while Friedreich’s ataxia and SCA3 have relatively robust and well-powered datasets, many SCAs and rarer hereditary ataxias lack sufficient longitudinal studies, limiting immediate trial readiness.

A critical challenge is the presymptomatic phase, where pathogenic processes may begin years before overt clinical dysfunction. Identifying biomarkers that can detect and track these early deviations—particularly in genetically confirmed but clinically asymptomatic individuals—will be central to advancing disease interception and to enabling therapeutic monitoring at the earliest stages.

The examples of NfL, polyQ-ATXN3, and the pons atrophy in SCA3, together with frataxin in Friedreich’s ataxia, illustrate how well-validated biomarkers can effectively bridge mechanistic insight and trial implementation. These biomarkers integrate molecular, structural, and functional readouts, reflecting convergent pathological pathways. However, validated target-engagement biomarkers remain limited for most ataxias, particularly in the context of emerging gene-targeted and RNA-based therapies. Such pharmacodynamic markers will be indispensable for assessing biological efficacy, dose selection, and potential off-target effects—even in ultra-rare genotypes.

Looking ahead, progress will rely on three converging fronts.

First, large multicenter longitudinal cohorts with standardized imaging, fluid, and digital protocols are needed to define disease trajectories, quantify progression rates, and establish clinically meaningful effect sizes. Initiatives such as ENIGMA-Ataxia, TRACK-FA, and EFACTS offer the structural foundation for such harmonization across diseases and biomarker platforms.

Second, clinical translation of molecular and gene-based therapies will require sensitive multimodal panels that integrate molecular signatures (e.g., mutant protein load, RNA species), cellular markers (e.g., NfL dynamics, mitochondrial dysfunction), and functional outputs (e.g., digital gait, postural-control and oculomotor metrics).

Third, advances in machine learning, multi-omics integration, and digital phenotyping provide unprecedented opportunities for individual-level prediction of progression and phenoconversion. Federated, interoperable data infrastructures will be essential to transition from fragmented datasets toward a scalable precision-medicine framework.

## Data Availability

No new data were created or analyzed in this study. Data sharing is not applicable to this article.

## Abbreviations

ADC: Apparent Diffusion Coefficient; AUC: Area Under the Curve; BARS: Brief Ataxia Rating Scale; BEST: Biomarkers, EndpointS, and other Tools (FDA); CANVAS: Cerebellar Ataxia with Neuropathy and Vestibular Areflexia Syndrome; CCFS: Composite Cerebellar Functional Severity; CGG: Cytosine–Guanine–Guanine (trinucleotide repeat); CI: Confidence Interval; CGM: Cortical Gray Matter; CNS: Central Nervous System; CSF: Cerebrospinal Fluid; CWM: Cerebral White Matter; DaT-SPECT: Dopamine Transporter Single-Photon Emission Computed Tomography; DRPLA: Dentatorubral–Pallidoluysian Atrophy; DTI: Diffusion Tensor Imaging; EFACTS: European Friedreich’s Ataxia Consortium for Translational Studies; ENIGMA: Enhancing NeuroImaging Genetics through Meta-Analysis (Ataxia working group); ESMI: European Spinocerebellar Ataxia/Machado–Joseph Disease Initiative; FA: Fractional Anisotropy; FACOMS: Friedreich’s Ataxia Clinical Outcome Measures Study; FDS: Functional Disability Stage; FDA: U.S. Food and Drug Administration; ^18^F-FDG-PET: Fluorodeoxyglucose Positron Emission Tomography; FRDA: Friedreich’s Ataxia; GAA: Guanine–Adenine–Adenine (trinucleotide repeat); GFAP: Glial Fibrillary Acidic Protein; HPO: Human Phenotype Ontology; HDAC: Histone Deacetylase; ICARS: International Cooperative Ataxia Rating Scale; ICP: Inferior Cerebellar Peduncle; ICC: Intraclass Correlation Coefficient; ^11^C-ITMM: ^11^C-Iodomethoxybenzyl-L-Thienylalanine; LC–MS: Liquid Chromatography–Mass Spectrometry; LOCA: Late-Onset Cerebellar Ataxia; MCP: Middle Cerebellar Peduncle; MD: Mean Diffusivity; mFARS: modified Friedreich’s Ataxia Rating Scale; mGluR1: Metabotropic Glutamate Receptor 1; MIBG: Meta-Iodobenzylguanidine; MRS: Magnetic Resonance Spectroscopy; MRI: Magnetic Resonance Imaging; NAA: N-Acetylaspartate; NfL: Neurofilament Light Chain; ONL: Outer Nuclear Layer; PD: Pharmacodynamic; PET: Positron Emission Tomography; PROM-Ataxia: Patient-Reported Outcome Measure for Ataxia; PROSPERO: International Prospective Register of Systematic Reviews; QA: Quality Assurance; QSM: Quantitative Susceptibility Mapping; QUADAS-2: Quality Assessment of Diagnostic Accuracy Studies-2; RD: Radial Diffusivity; READ score: Reading Eye Abnormality Digital score; RoB 2: Risk of Bias tool version 2; ROBINS-I: Risk Of Bias In Non-randomized Studies of Interventions; SARA: Scale for the Assessment and Rating of Ataxia; SCA: Spinocerebellar Ataxia; SCP: Superior Cerebellar Peduncle; Simoa: Single Molecule Array; SMC: Single-Molecule Counting; SOP: Standard Operating Procedure; SPG7: Spastic Paraplegia Type 7; SPECT: Single-Photon Emission Computed Tomography; SWiM: Synthesis Without Meta-analysis; TE: Target Engagement; TR-FRET: Time-Resolved Förster Resonance Energy Transfer; UCH-L1: Ubiquitin C-Terminal Hydrolase L1; VBM: Voxel-Based Morphometry; VOR: Vestibulo-Ocular Reflex; FXTAS: Fragile X-Associated Tremor/Ataxia Syndrome.
